# First-principles study of the structure, magnetism, and electronic properties of the all-Heusler alloy Co_2_MnGe/CoTiMnGe(100) heterojunction

**DOI:** 10.3389/fchem.2024.1434607

**Published:** 2024-07-09

**Authors:** Jianqiao He, Haishen Huang, Bo Wu, Guangxian Shen, Tingyan Zhou, Yuxin Gu, Lin Wen, Qingqing Zhang

**Affiliations:** ^1^ School of Physics and Electronic Science, Guizhou Normal University, Guiyang, China; ^2^ School of Physics and Electronic Science, Zunyi Normal University, Zunyi, China

**Keywords:** first-principles, Heusler alloy, magnetism, electronic properties, heterojunction

## Abstract

Based on first-principles calculations in the density functional theory, we systematically investigated the possible interface structure, magnetism, and electronic properties of the all-Heusler alloy Co_2_MnGe/CoTiMnGe(100) heterojunction. The calculation indicated that the Co_2_MnGe Heusler alloy is a half-metal with a magnetic moment of 4.97 μ_B_. CoTiMnGe is a narrow-band gap semiconductor and may act as an ultra-sensitive photocatalyst. We cannot find an “ideal” spin-polarization of 100% in CoCo termination and MnGe termination. Due to the interface interaction, the direct magnetic hybridization or indirect RKKY exchange will be weakened, leading to an increase in the atomic magnetic moment of the interfacial layer. For eight possible heterojunction structures, the half-metallic gaps in the Co_2_MnGe bulk have been destroyed by the inevitable interface states. The spin-polarization value of 94.31% in the CoCo-TiGe-B heterojunction revealed that it is the most stable structure. It is feasible to search for high-performance magnetic tunnel junction by artificially constructing suitable all-Heusler alloy heterojunctions.

## 1 Introduction

Magnetic tunnel junction (MTJ) is a kind of special spintronic device with a core “sandwich” three-layer film structure formed by inserting a layer of tunnel layer between two ferromagnetic layers (Wang et al., 2018; Penthorn et al., 2019; Song et al., 2019). It has been widely applied to magnetic read–write devices, such as magnetic sensors (Lenz and Edelstein, 2006), hard disks (Kent and Worledge, 2015), and magnetic random-access memory (Gallagher and Parkin, 2006). High tunnel magnetoresistance (TMR) ratios are a key parameter for the application of MTJs (Tao et al., 2014; Yin et al., 2018). When the spin-dependent current is perpendicular to the multilayer plane (Valet and Fert, 1993), the TMR ratio is strongly dependent on the spin-polarization of the ferromagnetic layer material. According to Jullière’s approximation (Jullière, 1975), the TMR ratio is calculated as Eq. 1:

$$\text{TMR}=\left|\frac{2P_{1}P_{2}}{1-P_{1}P_{2}}\right|\times 100\%,$$ (1)

where *P*_1_ and *P*_2_ are the spin-polarization ratios of two ferromagnetic layer materials, respectively. *P* is defined as (Jullière, 1975) Eq. 2:

$$P=\left|\frac{N_{↑}-N_{↓}}{N_{↑}+N_{↓}}\right|\times 100\%,$$ (2)

where *N*_↑_ and *N*_↓_ are the spin-up and spin-down state densities at the Fermi level. Therefore, the ferromagnetic layer material with a spin-polarization ratio of 100%, called a half-metallic material (de Groot et al., 1983), is regarded as an “ideal” electrode material for MTJ application.

Among many half-metallic candidate materials, the Heusler alloy family has attracted much attention, owing to high Curie temperature, extremely low Gilbert damping constant, and stable lattice match with common tunnel layer materials involving MgO and GaAs (Jourdan et al., 2014; Claudia et al., 2015; Wollmann et al., 2017). Many theoretical research studies revealed that many Heusler heterojunctions are promising candidates for MTJ application. In 2015, a theoretical study revealed that the Heusler alloy has a large TMR ratio of approximately 5,655% in the Co_2_MnAl/Ag/Co_2_MnAl spin valve (Li et al., 2015). In 2018, a considerably large TMR ratio of up to 2.8 × 10^6^% was found in the Ti_2_MnAl/InAs/Ti_2_MnAl(001) MTJ from the theoretical prediction (Han and Gao, 2018). However, for Ti_2_FeAl/GaAs(100) heterojunctions, first-principles studies showed that the TMR ratio is just close to 300% (Wu et al., 2019). Recently, Cui et al. (2021) studied the properties of double half-Heusler alloy Mn_2_CoCrZ_2_ (Z = *P*, As) with a GaAs semiconductor to construct an MTJ and obtained a TMR ratio as high as 7.96 × 10^8^%.

However, the oppressive fact is that the experimental TMR ratios are usually much less than theoretical results in Heusler alloy MTJs. In 2012, a TMR value of 1,995% in the full-Heusler MTJ Co_2_MnSi/MgO/Co_2_MnSi film was detected experimentally at low temperatures (Liu et al., 2012). For CoFeMnSi/MgO/CoFeMnSi films, the maximum TMR ratio is approximately 101% and 521% for complete B2 and partial L2_1_ ordered crystal structures at 10 K, respectively (Bainsla et al., 2018). As the temperature rises, many Heusler alloy TMR values will decline rapidly. Sakuraba et al. found TMR values of 570% at low temperature and 67% at room temperature in Co_2_MnSi/AlO/Co_2_MnSi tunnel junctions (Sakuraba et al., 2006). Marukame et al. (2007) found TMR values of 317% at low temperature and 109% at room temperature in the CoCrFeAl/MgO/CoFe tunnel junction. The physical and chemical mechanisms behind the temperature-dependent phenomena of TMR are very complex. A possible reason is that the spin-polarization of ferromagnetic materials is seriously damaged by the strong atomic electromagnetic interaction at the interface of the Heusler alloy and the semiconductor or insulator material. The inevitable interface effect, such as lattice mismatch, atomic disorder, atomic defect, lattice distortion, and structure reconstruction, will lead to decreasing TMR values with increasing temperature (Orgassa et al., 2000; Fang H. N. et al., 2020; Cheng et al., 2021; Hirohata et al., 2022). It is easy to assume that reducing the interface mismatch between the electrode and the barrier layer will be a feasible method for increasing TMR ratios. The all-Heusler alloy heterojunction, which is created by the ferromagnetic material and the barrier layer from Heusler alloys with very similar lattice structures, has an extremely low lattice mismatch ratio and can preserve the atomic potential periodicity to the maximum extent in the bulk of the Heusler ferromagnetic material. In many previous studies for all-Heusler alloy MTJs, an extremely high TMR value has been detected. In an early study on all-Heusler alloy MTJ Co_2_CrSi/Cu_2_CrAl/Co_2_CrSi, spin-polarization of nearly 100% could be reserved in the ferromagnetic layer (Bai et al., 2012). In 2021, Pradines et al. (2021) studied two all-Heusler alloy MTJs, Co_2_MnSi/Fe_2_TiSi (001) and Co_2_MnSi/Fe_2_VAl (001), and showed that the half-metallicity of the Fe_2_/MnSi-terminated interface even withstands atomic intermixing. In 2022, Feng et al. (2022) also studied the all-Heusler alloy MTJ CoFeTiSi/Fe_2_TiSi/CoFeTiSi system, and a high TMR ratio of 2.4 × 10^8^% was predicted. In 2023, Bhattacharya and Chakrabarti (2023) found a high TMR value of the order of 10^6^ in MTJ Co_2_MnSb/TiCoSb/Co_2_MnSb using electronic structure calculations based on the density flood theory. By investigating theoretically all-Heusler alloy MTJ Co_2_MnSi/Ni_2_NiSi/Co_2_MnSi, Bai et al. (2013) suggested future theoretical and experimental efforts in developing high-performance all-Heusler current-perpendicular-to-plane giant magnetoresistance junctions for the read heads of next-generation high-density hard disk drives.

To deeply understand the interface physical and chemical mechanism in the all-Heusler alloy heterojunction and find out more high-performance Heusler MTJs, an all-Heusler alloy MTJ Co_2_MnGe/CoTiMnGe(100) heterojunction was constructed in our work. The structure, magnetism, and interface electronic properties have been systematically investigated from the first principles. The partial density of states (PDOS) and spin-polarization of eight possible interface terminations have been calculated. This work will further confirm that it is feasible to search for high-performance MTJs by artificially constructing suitable all-Heusler alloy heterojunctions.

## 2 Computational details

**FIGURE 1:**
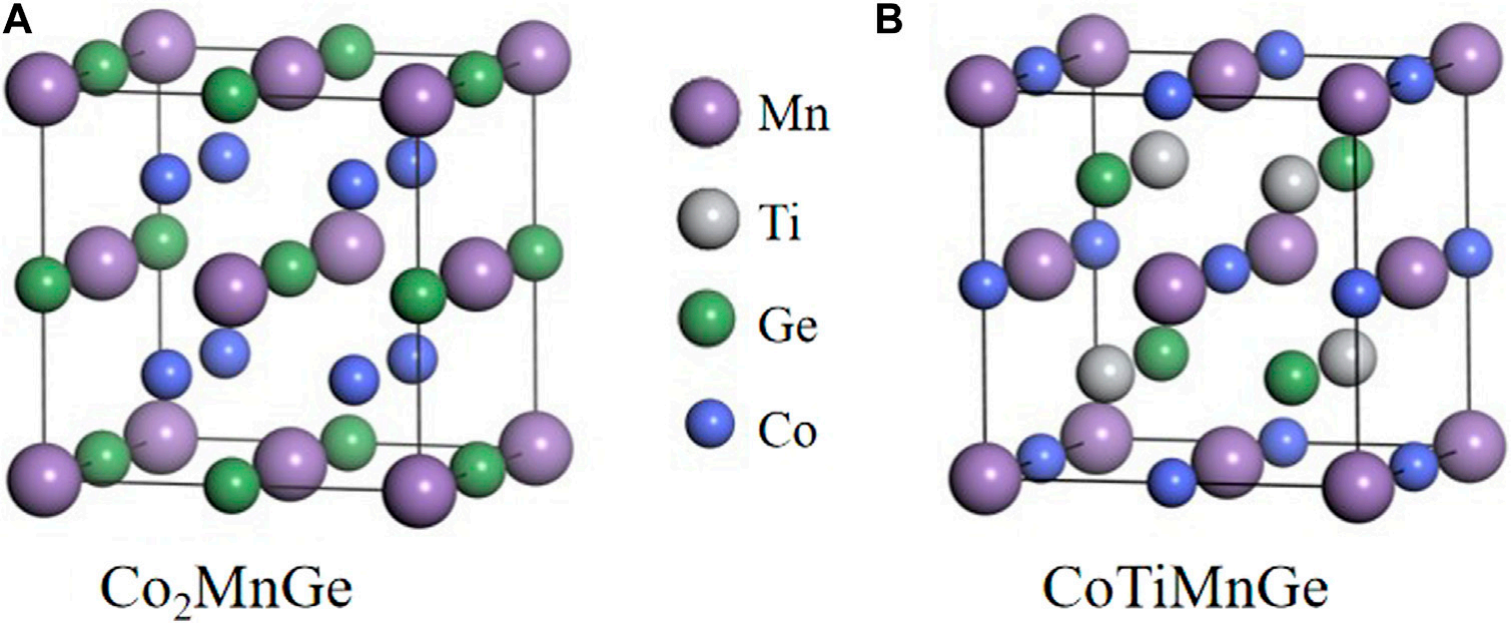
Crystal structure of **(A)** Co_2_MnGe and **(B)** CoTiMnGe.

**FIGURE 2:**
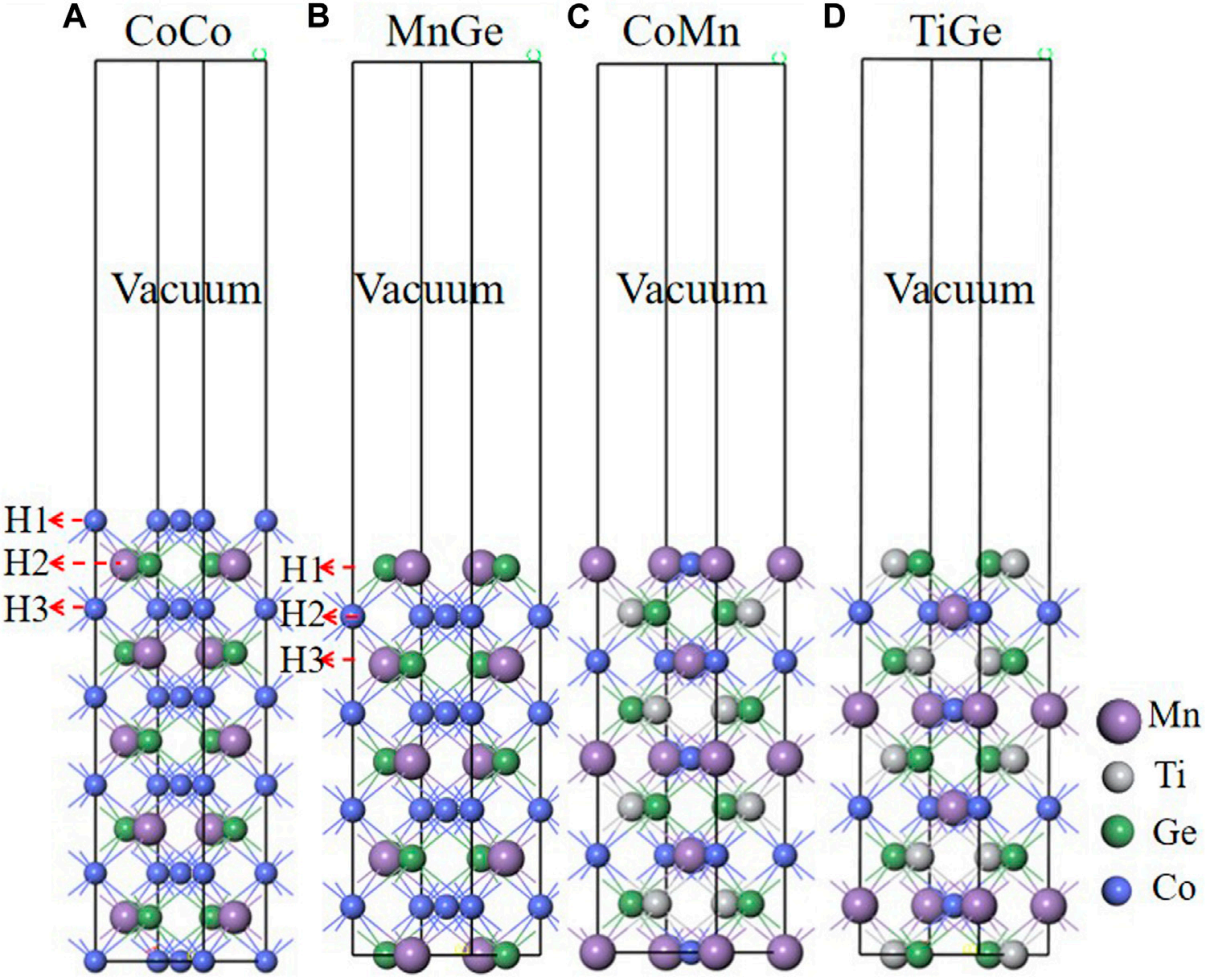
**(A)** CoCo termination and **(B)** MnGe termination of the Co_2_MnGe(100) surface. **(C)** CoMn termination and **(D)** TiGe termination of the CoTiMnGe(100) surface.

**FIGURE 3:**
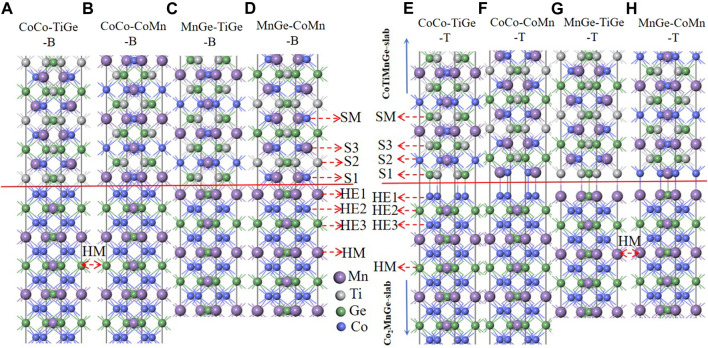
Eight Co_2_MnGe/CoTiMnGe(100) heterojunctions. **(A)** CoCo-TiGe-B, **(B)** CoCo-CoMn-B, **(C)** MnGe-TiGe-B, **(D)** MnGe-CoMn-B, **(E)** CoCo-TiGe-T, **(F)** CoCo-CoMn-T, **(G)** MnGe-TiGe-T, and **(H)** MnGe-CoMn-T. The interface layer, the subinterface layer, the next subinterface layer, and the middle layer in the Co_2_MnGe alloy are marked as HE1, HE2, HE3, and HM, respectively. In the CoTiMnGe slab, the interface layer, the subinterface layer, the next subinterface layer, and the middle layer are marked as S1, S2, S3, and SM, respectively.

First, we constructed the Co_2_MnGe and CoTiMnGe crystal structures, as shown in Figure 1. The space group of the full-Heusler alloy Co_2_MnGe is FM-3M (Kono et al., 2019), and the Co atoms are located at (0.25, 0.25, 0.25) and (0.75, 0.75, 0.75), Mn atoms at (0, 0, 0), and Ge atoms at (0.5, 0.5, 0.5). The space group of the LiMgPbSb-type Heusler alloy CoTiMnGe is F-43M, and in its unit cell, the Co atoms are located at (0.5, 0.5, 0.5), Ti atoms at (0.75, 0.75, 0.75), Mn atoms at (0, 0, 0), and Ge atoms at (0.25, 0.25, 0.25). First, the Co_2_MnGe and CoTiMnGe bulks are optimized to find their lowest energy structures. For the optimized structure, two atomic terminations of 11 and 9 layers for Co_2_MnGe (100) and 9 layers for CoTiMnGe (100) are cut along the Miller index [100] crystal direction, respectively. Four possible surface supercells, including 11 layers of CoCo termination and 9 layers of MnGe, CoMn, and TiGe terminations are shown in Figure 2. Considering the top and bridge atomic connecting types at the interface, the eight possible interface heterojunctions, which are named CoCo-TiGe-B, CoCo-CoMn-B, MnGe-TiGe-B, MnGe-CoMn-B, CoCo-TiGe-T, CoCo-CoMn-T, MnGe-TiGe-T, and MnGe-CoMn-T according to atoms and their connecting type from Co_2_MnGe and CoTiMnGe surface terminations, are established as shown in Figure 3. For the convenience of discussions, the interface, the subinterface, the next subinterface, and the middle layer in the Co_2_MnGe slab are marked as HE1, HE2, HE3, and HM, respectively. In the CoTiMnGe slab, the interface, the subinterface, the next subinterface, and the middle layer are marked as S1, S2, S3, and SM, respectively.

All calculations are implemented by the VASP simulation package based on the density functional theory (DFT) (Blöchl, 1994; Wang et al., 2021). The generalized gradient approximation (GGA) is used to describe the interactions between the exchange relationships (Perdew et al., 1996). In all calculations, the Vanderbilt-type supersoft pseudopotential (Vanderbilt, 1990) and the valence electronic configurations of Mn (d^6^s^1^), Co (d^8^s^1^), Ge (s^2^p^2^), and Ti (d^3^s^1^) are used. In the process of calculating the structural optimization and properties of the bulk, all alloys are first assumed to be ferromagnetic, and spin-polarization is set for each structure. *k*-points in the Brillouin zone of 11 × 11 × 11, a self-consistent convergence criterion of 10^–6^ eV/atom, a cutoff energy of 420 eV, and a maximal force convergence criterion of 0.02 eV/Å are applied, respectively. The choice of k-points and cutoff energy is to ensure that the total energy converges to within 2 × 10^−3^ eV per atom. For the computation of Co_2_MnGe(100) surfaces and heterojunctions, the parameters are identical to the block settings, except for k-points in the Brillouin zone of 11 × 11×1. All calculation parameters have been tested carefully to ensure the accuracy of the results.

## 3 Results and discussion

### 3.1 Structure, magnetism, and electronic properties in bulks

**FIGURE 4:**
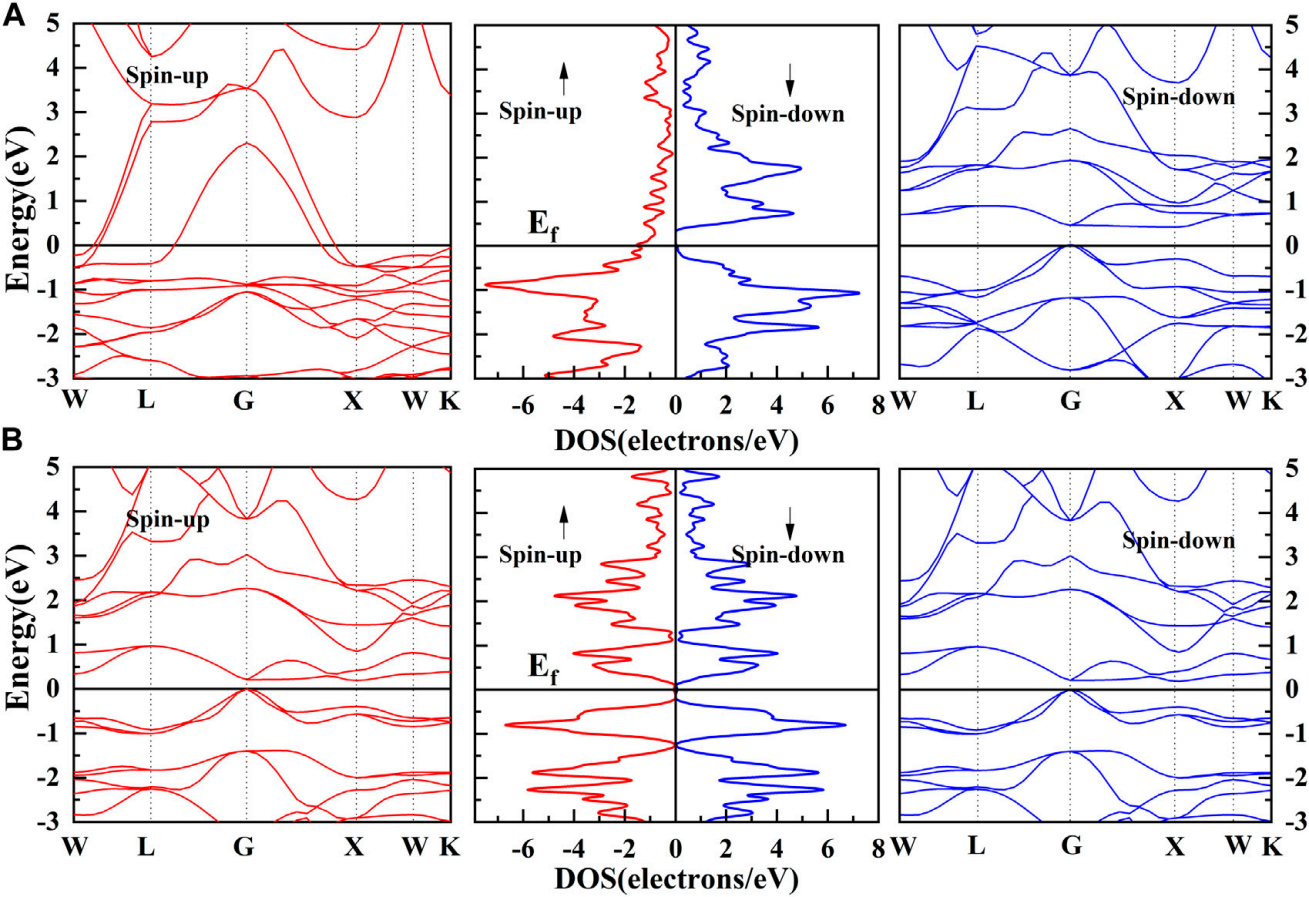
Band structure and DOS of **(A)** Co_2_MnGe bulk and **(B)** CoTiMnGe bulk.

By structure optimization to the Heusler alloy Co_2_MnGe and CoTiMnGe bulks, their crystal structures with the lowest energy can be obtained. After the relaxation of the bulk Co_2_MnGe, we obtain an equilibrium lattice constant of 5.742 Å, which is very close to both the theoretical value of 5.755 Å (Han et al., 2017) and the experimental value of 5.747 Å (Ouardi et al., 2011). The lattice constant of CoTiMnGe in the ground state is 5.817 Å. Hence, the lattice mismatch ratio of the Heusler alloy Co_2_MnGe/CoTiMnGe heterojunction is less than 0.65%. For Co_2_MnGe, the calculated total magnetic moment is 4.97 μ_B_ in a unit cell, which is consistent with the previous research (Han et al., 2017; Özduran et al., 2020), and the atomic magnetic moment of the Co1, Co_2_, Mn, and Ge atoms is 1.00, 1.00, 3.01, and−0.04 μ_B_, respectively. CoTiMnGe is a semiconductor because of a total magnetic moment of zero. It is consistent with the Slater–Pauling law of *M*_t_ = *Z*_t_-24 well (Graf et al., 2011). Band structures and density of states (DOS) of the Co_2_MnGe and CoTiMnGe bulks are shown in Figure 4. For the Co_2_MnGe bulk, as shown in Figure 4A, the spin-up channel crosses the Fermi level and shows metal behaviors, while the spin-down channel presents a semiconductor gap. So, the Heusler alloy Co_2_MnGe bulk is a typical half-metal material with a half-metallic gap of approximately 0.435 eV. For the CoTiMnGe bulk, as shown in Figure 4B, the symmetric band structures and DOS show that the alloy is a narrow-band gap semiconductor with a bandwidth of 0.2 eV. It can serve as an ultra-sensitive photocatalyst to absorb a wide range of sunlight. A narrow band gap enhances the photocatalyst’s light-harvesting ability; however, it can lead to electron–hole recombination. This issue can be mitigated through elemental doping, metal modification, and the construction of novel heterojunctions (Fang Y. et al., 2020; Zhu et al., 2021; Reddy et al., 2022). We found that the calculated Co_2_MnGe band gap is similar to that of previous studies (Sharma and Pandey, 2014), which indicates that our calculation method is reasonably reliable.

### 3.2 Surface properties of the Heusler alloy Co_2_MnGe(100)

In order to further understand the surface characteristics in the vacuum, the atomic relaxation, atomic magnetic moments, and PDOS of the Co_2_MnGe(100) surfaces are calculated. The surface, the subsurface, and the next subsurface layer are marked as H1, H2, and H3, respectively, in two possible atomic terminations for the sake of discussion, as shown in Fig 2 (a) and (b). In Table 1, the atomic displacements of the outermost three layers in CoCo termination and MnGe termination in the Co_2_MnGe(100) surface are listed. The positive and negative values (in parentheses) indicate that the corresponding atoms move toward the vacuum and the slab, respectively. In CoCo termination, both the Co atoms at the H1 and the Mn atoms at the H2 layer extend into the vacuum layer, while the Ge atoms at the H2 shrink inside. However, in MnGe termination, all of the Co, Mn, and Ge atoms at the H2 extend to the vacuum. The largest atomic displacement comes from the Mn atoms at the H1. As a result, MnGe termination exhibits a roughness due to different atomic relaxation.

**TABLE 1:** Atomic displacements (in unit of Å) of the interface (H1), the subinterface (H2), and the next subinterface layer (H3) in CoCo termination and MnGe termination of the Co_2_MnGe(100) surface. The positive and negative values indicate that the corresponding atoms move toward the vacuum and the slab, respectively.

Termination	H1	H2	H3
CoCo	Co1(0.001), Co2(0.018)	Mn(0.016), Ge(-0.024)	Co1(0.006), Co2(0.007)
MnGe	Mn(0.293), Ge(0.031)	Co1(0.047), Co2(0.048)	Mn(0.037), Ge(-0.053)

In Table 2, the atomic magnetic moments of the outermost three layers in CoCo termination and MnGe termination in the Co_2_MnGe(100) surface are also listed. Owing to the reduction of atomic coordination numbers at the surfaces, the crystal field is weakened, and the localization from the outermost layer atoms is enhanced. As a result, the magnetic moment of the surface atoms improves significantly compared with the corresponding value in the bulk. Similar surface behaviors are also observed in Co_2_FeSi(100) (Khosravizadeh et al., 2009) and Co_2_MnSi(001) (Hashemifar et al., 2005). For Co and Mn atoms at H2 and H3, their atomic moments are reduced compared to the value in the bulk except for the Mn atoms at H3. The complex magnetic properties may originate from competition between direct magnetic exchange from localizations and RKKY indirect magnetic interaction from atomic displacements. When the distance between the H2 and H3 layers is narrowed, the negative sp atomic magnetic moments of Ge will increase. It can lead to increase in the RKKY magnetic effects. In the RKKY indirect exchange mechanism, the sp-conducting electrons can serve as a “bridge” for local d–electron interactions (Wu et al., 2010). This sp–d hybrid interaction always causes an antiferromagnetic order of the sp atoms and transit metal atoms, resulting in a negative magnetic moment contribution.

**TABLE 2:** Atomic magnetic moment (in unit of μ_B_) of the interface (H1), the subinterface (H2), and the next subinterface layer (H3) in CoCo termination and MnGe termination of the Co_2_MnGe(100) surface.

Termination	H1	H2	H3
CoCo	Co1(1.170), Co2(1.245)	Mn(2.898), Ge(-0.065)	Co1(0.875), Co2(0.881)
MnGe	Mn(3.646), Ge(-0.097)	Co1(0.824), Co2(0.906)	Mn(3.079), Ge(-0.054)

**FIGURE 5:**
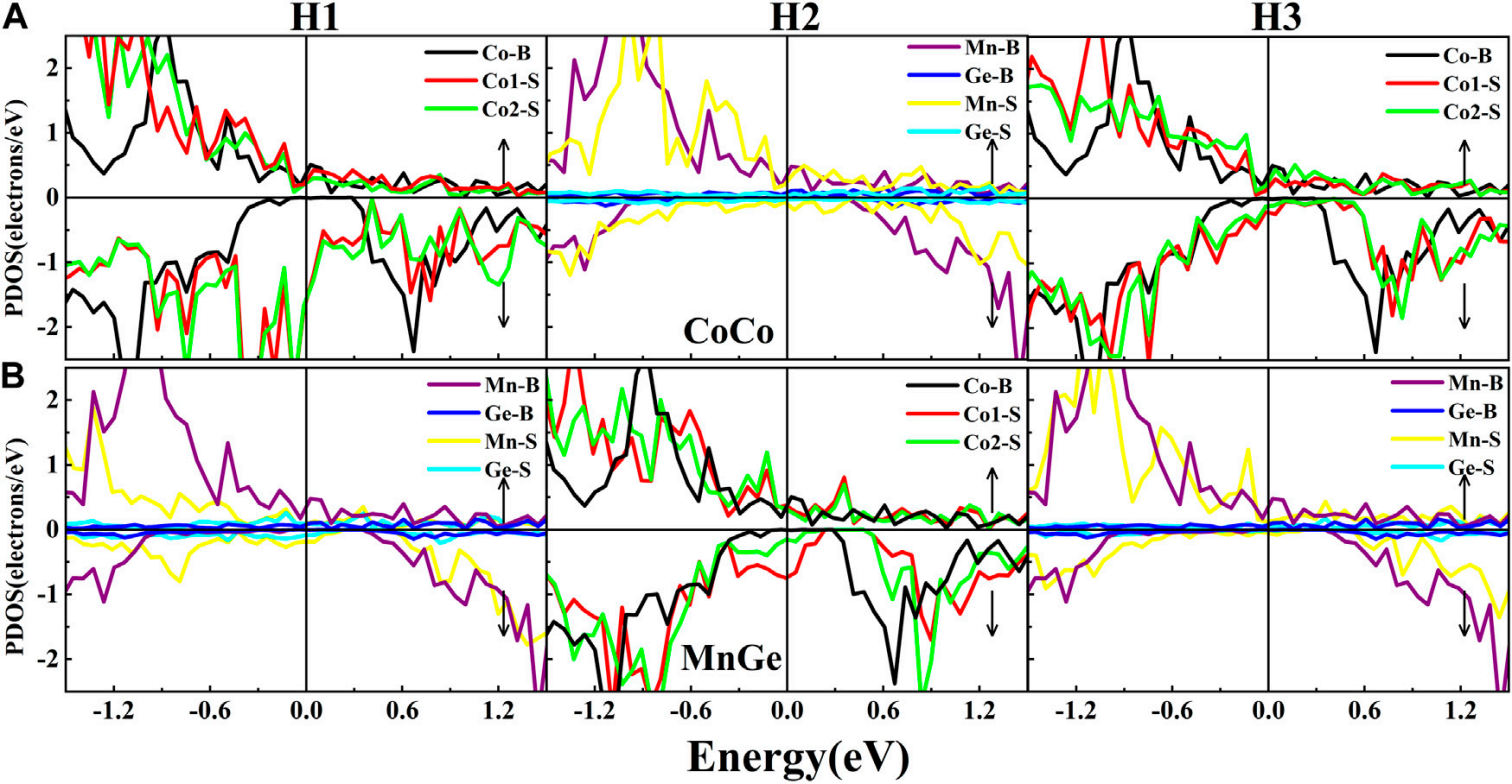
Atomic PDOS at the surface layer (H1), the subsurface layer (H2), and the next subsurface layer (H3) in **(A)** CoCo termination and **(B)** MnGe termination of the Co_2_MnGe (100) surface. B represents the bulk, and S represents the surface.

In Figure 5, the PDOS of the outermost three layer atoms in CoCo and MnGe terminations are shown. In the CoCo termination, for Co atoms at H1 and H3, the half-metallic gap in the bulk has been occupied by the surface states. The spin-down gap of Mn at H2 also suffers a slight reduction. In the MnGe termination, several surface states from Mn or Ge atoms at H1 appear in the half-metallic gap. At H2, the DOS of Co atoms have damaged the half-metallicity severely. All possible terminations in the Co_2_MnGe(100) surface cannot reserve the “ideal” spin-polarization of 100%, owing to existence of the surface states.

### 3.3 Interface structures and magnetic properties in Co_2_MnGe/CoTiMnGe(100)

An effective way to improve the spin-polarization of ferromagnetic layer materials in MTJ, as mentioned in the Introduction section, is to construct an all-Heusler alloy system with a structurally similar insulating layer material connected to it. Here, we will focus on an all-Heusler alloy Co_2_MnGe/CoTiMnGe(100) heterojunction. In Table 3, the atomic displacements in parentheses at the outermost layers in the eight possible heterojunctions are listed. The positive or negative value indicates the shift toward the interface layer or the interior layer in the Heusler alloy compared with the atomic position in the bulk, respectively.

**TABLE 3:** Atomic displacements (in unit of Å) of the outermost layers in the eight possible heterojunctions. The positive or negative value indicates the shift toward the interface layer or the interior layer in the Heusler alloy compared with the atomic position in the bulk, respectively. Here, H1, H2, and H3 are the interface, the subinterface, and the next interface layer in the Co_2_MnGe slab, respectively; and S1, S2, and S3 are the interface, the subinterface, and the next interface layer in the CoTiMnGe slab, respectively.

Heterojunction	HE1	HE2	HE3	S1	S2	S3	Atomic bond type at the interface	Bond length/Å
CoCo-CoMn-T	Co1(-0.086) Co2(0.349)	Mn(0.170) Ge(0.106)	Co1(0.080) Co2(0.349)	Co(0.126) Mn(0.598)	Ti(0.131) Ge(0.130)	Co(0.077) Mn(0.085)	Co1-Co Co2-Mn	2.231 2.196
CoCo-CoMn-B	Co1(0.018) Co2(0.001)	Mn(0.016) Ge(-0.025)	Co1(-0.006) Co2(-0.007)	Co(0.114) Mn(0.261)	Ti(0.058) Ge(0.050)	Co(0.042) Mn(0.036)	Co1-Co Co1-Mn Co2-Co Co2-Mn	2.529 2.445 2.540 2.455
CoCo-TiGe-T	Co1(0.030) Co2(0.067)	Mn(0.121) Ge(0.120)	Co1(0.032) Co2(0.068)	Ti(0.329) Ge(0.071)	Co(0.084) Mn(0.143)	Ti(0.065) Ge(0.077)	Co1-Ti Co2-Ge	2.125 2.347
CoCo-TiGe-B	Co1(0.029) Co2(0.054)	Mn(-0.008) Ge(-0.043)	Co1(-0.005) Co2(-0.008)	Ti(0.072) Ge(0.024)	Co(0.030) Mn(0.048)	Ti(0.016) Ge(0.023)	Co1-Ti Co1-Ge Co2-Ti Co2-Ge	2.507 2.535 2.521 2.549
MnGe-CoMn-T	Mn(0.083) Ge(0.234)	Co1(0.096) Co2(0.100)	Mn(0.069) Ge(0.046)	Co(0.127) Mn(0.482)	Ti(0.116) Ge(0.117)	Co(0.082) Mn(0.083)	Mn - Mn Ge-Co	2.204 2.406
MnGe-CoMn-B	Mn(0.012) Ge(0.107)	Co1(-0.003) Co2(0.000)	Mn(-0.017) Ge(-0.008)	Co(0.054) Mn(0.146)	Ti(0.028) Ge(0.000)	Co(0.025) Mn(0.008)	Mn-Co Mn - Mn Ge-Co Ge-Mn	2.538 2.485 2.591 2.534
MnGe-TiGe-T	Mn(0.281) Ge(0.030)	Co1(0.047) Co2(0.048)	Mn(0.037) Ge(0.052)	Ti(0.265) Ge(0.079)	Co(0.100) Mn(0.142)	Ti(0.054) Ge(0.064)	Mn-Ge Ge-Ti	2.611 2.677
MnGe-TiGe-B	Mn(0.259) Ge(-0.039)	Co1(0.103) Co2(0.015)	Mn(0.019) Ge(0.049)	Ti(0.258) Ge(0.035)	Co(0.036) Mn(0.151)	Ti(0.035) Ge(0.057)	Mn-Ti Mn-Ge Ge-Ti Ge-Ge	2.691 2.823 2.877 3.057

Altogether, we can find that atomic displacement values at the outermost layers will decrease significantly as their positions are closer to the middle layer. It indicates that the interfacial interaction decreases as the atoms move closer to the middle layer. Owing to lattice matching and continuity of the atomic periodic potential at the interface constructed by the Heusler ferromagnetic alloy Co_2_MnGe and the Heusler semiconducting alloy CoTiMnGe, all displacements are relatively small values. Most displacements are approximately 0.5 Å from the Co atom at HE1 and the Mn atom at S1 in the CoCo-CoMn-T heterojunction. A few displacement values of approximately 0.2–0.3 Å appear from interface atoms in MnGe-CoMn-T, CoCo-CoMn-B, and MnGe-TiGe-B systems. In the four remaining heterojunctions, the interface atoms suffer relatively small shifts. In addition, those atoms at HE2 or S2 and HE3 or S3 have an extremely small movement in the original location. Moreover, compared with the “top” atomic connecting style, the spacing distance between the Heusler alloy Co_2_MnGe and the CoTiMnGe slab is relatively smaller in the heterojunction, and the interfacial atomic bond length is relatively larger. As a result, the interaction between interfacial atoms is weaker, and the interfacial atomic displacement is smaller. It should be mentioned that the atomic displacement at the outermost layer is the result of the Coulomb interactions among atoms. A balance of forces causes atoms to move relative to each other.

The magnetic properties of interfacial atoms usually depend on the competition between d-electronic localization and delocalization from transition metal atoms, as well as other indirect magnetic exchanges, such as the RKKY mechanism. We calculated the atomic-resolved magnetic moments of the interface HE1, the subinterface HE2, the next interface HE3, and the middle layer HM in the Co_2_MnGe slab, as well as the interface S1, the subinterface S2, the next interface S3, and the middle SM layer in the CoTiMnGe slab, as listed in Table 4. It can be found that the atomic magnetic moment at the middle layer HM or SM is very close to its bulk value, which shows that our computational method can deal with the models reasonably. Due to a small atomic displacement at the inner layers, such as HE2 or S2 and HE3 or S3, the atomic magnetic moment slightly varies around its value in the bulk. For Mn and Ti atoms at the S1 layer from the CoTiMnGe slab, because of a strong atomic interaction, interface atoms have been spin-polarized. In particular, the magnetic atoms at the S1 layer have a large magnetic moment. In eight Co_2_MnGe/CoTiMnGe heterojunctions, due to the fact that the interface atomic bond length will be increased compared with the value in the bulk because of the interface interaction, the direct magnetic hybridization or indirect RKKY exchange will be weakened.

**TABLE 4:** Atom-resolved magnetic moments (in unit of μ_B_) at the interface (HE1), the subinterface (HE2), the next interface (HE3), and the middle layer (HM) in the Co_2_MnGe slab and the interface (S1), the subinterface (S2), the next interface (S3), and the middle layer (SM) in the CoTiMnGe slab.

Heterojunction	HE1	HE2	HE3	HM	S1	S2	S3	SM
CoCo-CoMn-T	Co1(1.523) Co2(-0.015)	Mn(2.917) Ge(-0.056)	Co1(0.768) Co2(0.822)	Mn(3.121) Ge(-0.051)	Co(0.805) Mn(3.385)	Ti(-0.254) Ge(-0.027)	Co(0.022) Mn(-0.154)	Co(-0.022) Mn(0.013)
CoCo-CoMn-B	Co1(1.504) Co2(1.388)	Mn(3.125) Ge(-0.058)	Co1(0.976) Co2(0.966)	Mn(3.157) Ge(-0.049)	Co(1.434) Mn(2.937)	Ti(-0.264) Ge(-0.022)	Co(0.121) Mn(-0.044)	Co(0.001) Mn(0.013)
CoCo-TiGe-T	Co1(-0.056) Co2(-0.188)	Mn(2.981) Ge(-0.036)	Co1(0.926) Co2(0.793)	Mn(3.069) Ge(-0.051)	Ti(-0.064) Ge(-0.012)	Co(-0.011) Mn(0.200)	Ti(-0.028) Ge(-0.002)	Ti(-0.010) Ge(-0.001)
CoCo-TiGe-B	Co1(0.895) Co2(0.961)	Mn(3.280) Ge(-0.047)	Co1(1.050) Co2(0.983)	Mn(3.159) Ge(-0.049)	Ti(-0.117) Ge(-0.006)	Co(0.130) Mn(-0.099)	Ti(0.024) Ge(0.000)	Ti(-0.007) Ge(0.007)
MnGe-CoMn-T	Mn(2.955) Ge(-0.061)	Co1(0.970) Co2(0.974)	Mn(3.094) Ge(-0.051)	Mn(3.106) Ge(-0.050)	Co(0.332) Mn(3.032)	Ti(-0.187) Ge(-0.025)	Co(-0.031) Mn(-0.014)	Co(-0.008) Mn(-0.030)
MnGe-CoMn-B	Mn(3.127) Ge(-0.005)	Co1(1.042) Co2(1.058)	Mn(3.172) Ge(-0.046)	Mn(3.176) Ge(-0.053)	Co(0.802) Mn(-1.454)	Ti(0.105) Ge(0.011)	Co(-0.080) Mn(0.119)	Co(0.023) Mn(-0.004)
MnGe-TiGe-T	Mn(3.624) Ge(-0.062)	Co1(0.845) Co2(0.891)	Mn(3.080) Ge(-0.055)	Mn(3.043) Ge(-0.053)	Ti(0.000) Ge(-0.028)	Co(0.005) Mn(-0.012)	Ti(0.005) Ge(0.001)	Ti(0.002) Ge(0.000)
MnGe-TiGe-B	Mn(3.187) Ge(-0.029)	Co1(0.843) Co2(1.021)	Mn(3.132) Ge(-0.053)	Mn(3.094) Ge(-0.053)	Ti(-0.409) Ge(-0.026)	Co(-0.138) Mn(0.282)	Ti(-0.031) Ge(-0.002)	Ti(-0.006) Ge(0.000)

It can be the result of the increase in the atomic magnetic moment at the HE1 or S1 layer. However, in the CoCo-CoMn-T supercell, the Co atom at the HE1 and the Mn atom at the S1 move outside the slab and are close to each other, and the spacing distance between the HE1 and HE2 layers gets smaller. The Co atomic moment at the HE1 will also be smaller, owing to d-electronic localization. A similar magnetic mechanism appears in the CoCo-TiGe-T junction. For the rest atomic magnetic properties at the inner layer involving HE2 or S2 and HE3 or S3, the magnetic mechanism is very complex. Direct and indirect RKKY magnetic exchange, which depends on the environment around the atoms, plays an important role in atom magnetic properties.

### 3.4 Interface electronic properties and spin-polarization in Co_2_MnGe/CoTiMnGe(100)

**FIGURE 6:**
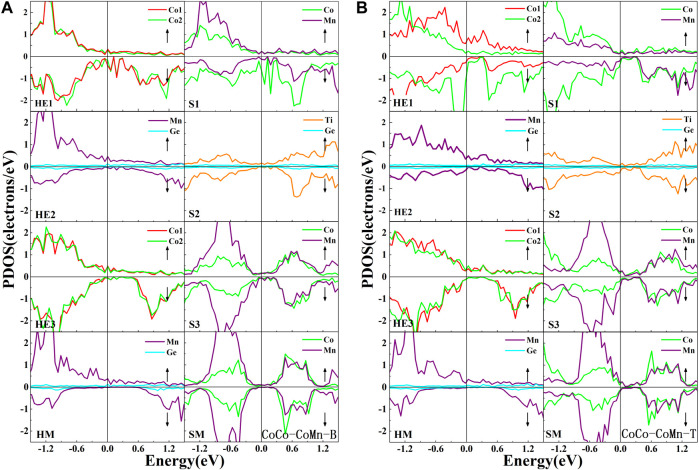
Atomic PDOS of the interface layer (HE1), the subinterface layer (HE2), the next subinterface layer (HE3), and the middle layer (HM) in the Co_2_MnGe slab and the interface layer (S1), the subinterface layer (S2), the next interface layer (S3), and the middle layer (SM) in the CoTiMnGe slab of **(A)** CoCo-CoMn-B and **(B)** CoCo-CoMn-T heterojunctions.

In order to study the electronic properties of the interface layer atoms, we calculated the atomic PDOS at the outermost three interface layers and the middle layer from the Co_2_MnGe and CoTiMnGe slabs, respectively, see Figure 6 for details. Moreover, the spin-polarization *P*, spin-up state density $N_{↑}$ , and spin-down state density $N_{↓}$ at the Fermi level at the outermost layers in eight heterojunctions were also calculated. Here, I-type includes the interface layer HE1 and the subinterface layer HE2 in the Co_2_MnGe slab, while II-type includes the interface layer S1 in the CoTiMnGe slab and I-type atomic layers. In eight Co_2_MnGe/CoTiMnGe(100) heterojunctions, the PDOS of the middle layer atoms is consistent with the shape of the bulk. The more the atom is close to the middle layer, the closer its PDOS morphology is to the middle. For eight possible heterojunction structures, the half-metallic gaps in the Co_2_MnGe bulk have been destroyed by the inevitable interface states. We cannot find an “ideal” spin-polarization of 100% from the I-type or II-type structure in the Co_2_MnGe/CoTiMnGe(100) heterojunctions.

As shown in Figure 6A, in the CoCo-CoMn-B structures, owing to the fierce interaction between the Co atom at the HE1 layer and the Mn atom at the S1 layer, the half-metallic or semiconductor properties in the Co_2_MnGe or CoTiMnGe bulk have been damaged. A few interface states appear in the half-metallic gap from Mn and Co atoms at the HE2 or HE3 layer in the Co_2_MnGe slab. It further reduced the spin-polarization of the ferromagnetic layer. The spin-polarized ratio in I-type and II-type structures is listed in Table 5. It can be found that a relatively low spin-polarization value of approximately 5.68% is detected in the CoCo-CoMn-B system. For the CoCo-CoMn-T structure, as shown in Figures 6A, B, large depolarization contribution comes from Co atoms at the HE1 and S1 layers. The spin-polarization value of the system is less than 8.8%.

**TABLE 5:** Spin-polarization *P*, spin-up state density $N_{↑}$ , and spin-down state density $N_{↓}$ at the Fermi level at the outermost layers in eight heterojunctions. Here, I-type includes the interface layer (HE1) and the subinterface layer (HE2) in the Co_2_MnGe slab, while II-type includes the interface layer (S1) in the CoTiMnGe slab and I-type atomic layers.

Interface layer	I-type			II-type		
	*N*_↑_(states/eV)	*N*_*↓*_(states/eV)	*P* (%)	*N*_↑_(states/eV)	*N*_*↓*_(states/eV)	*P* (%)
CoCo-CoMn-T	1.358	1.030	13.74	1.724	1.445	8.80
CoCo-CoMn-B	1.179	0.659	28.29	1.636	1.460	5.68
CoCo-TiGe-T	2.567	1.484	26.73	2.735	1.987	15.84
CoCo-TiGe-B	1.055	0.029	94.65	1.333	0.039	94.31
MnGe-CoMn-T	1.419	1.004	17.13	1.921	1.949	0.72
MnGe-CoMn-B	1.121	0.026	95.47	2.642	0.030	97.75
MnGe-TiGe-T	0.907	0.855	2.95	1.106	1.398	11.66
MnGe-TiGe-B	1.055	0.726	18.47	1.287	0.987	13.19

**FIGURE 7:**
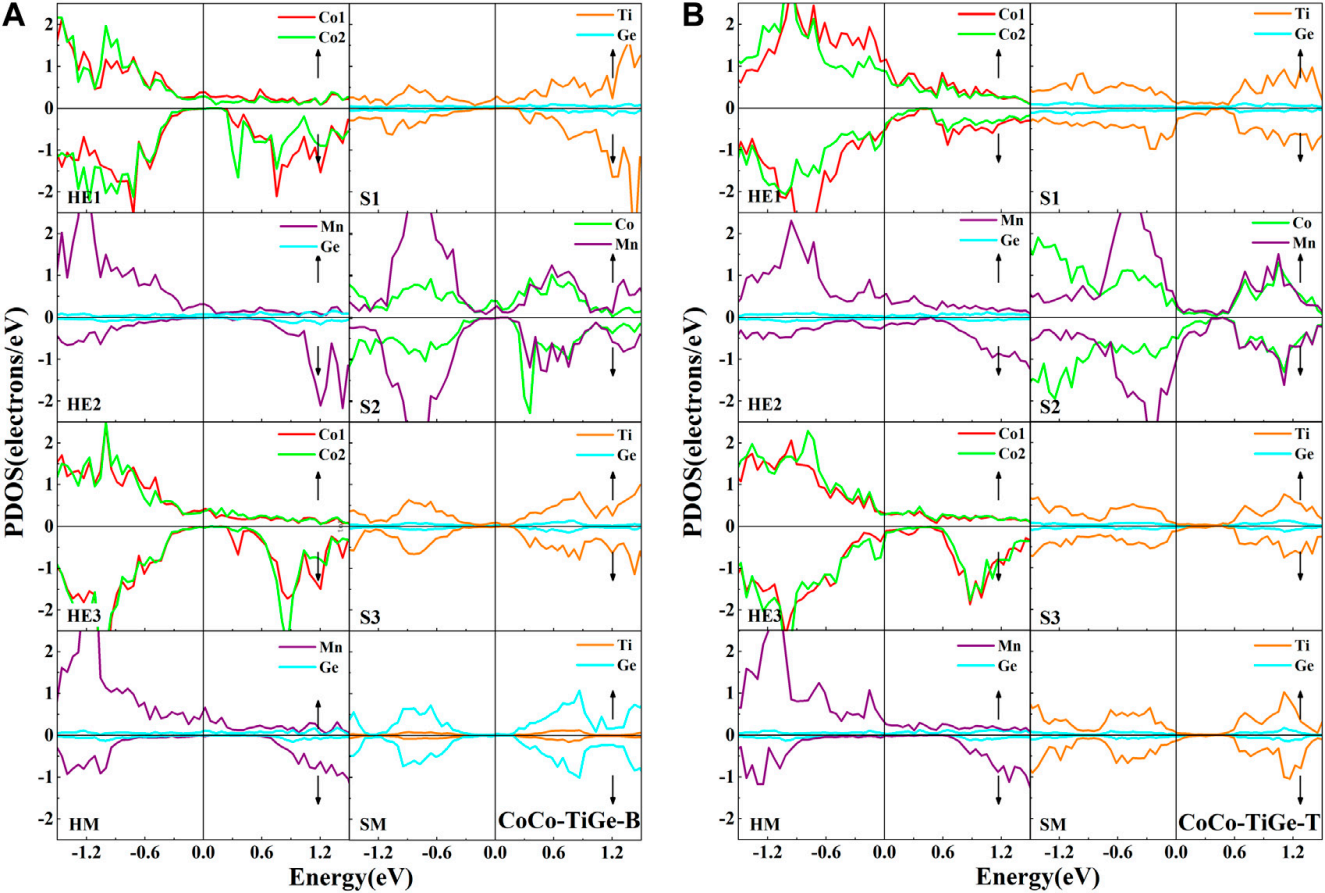
Atomic PDOS of the interface layer (HE1), the subinterface layer (HE2), the next subinterface layer (HE3), and the middle layer (HM) in the Co_2_MnGe slab and the interface layer (S1), the subinterface layer (S2), the next interface layer (S3), and the middle layer (SM) in the CoTiMnGe slab of **(A)** CoCo-TiGe-B and **(B)** CoCo-TiGe-T heterojunctions.

In the CoCo-TiGe-B heterojunction, as shown in Figure 7A, it is observed that the half-metallic properties in the bulk are preserved for the Co atom at the HE1 layer and the Mn atom at the HE2 layer. The semiconductor gap in the CoTiMnGe bulk is also retained. Only a few peaks from the Co atoms at the HE3 layer appear in the spin-down gap. It causes a slight decrement in spin-polarization. As a result, the spin-polarization value of 94.31% in II-type structures is detected. For the CoCo-TiGe-T heterojunction, as shown in Figure 7B, a strong interaction between interface atoms, Ti atoms at the S1, Co and Mn atoms at the S2, leads to strong spin polarization. We obtain a small spin-polarization value of 15.8% in the II-type structure from the CoCo-TiGe-T heterojunction.

**FIGURE 8:**
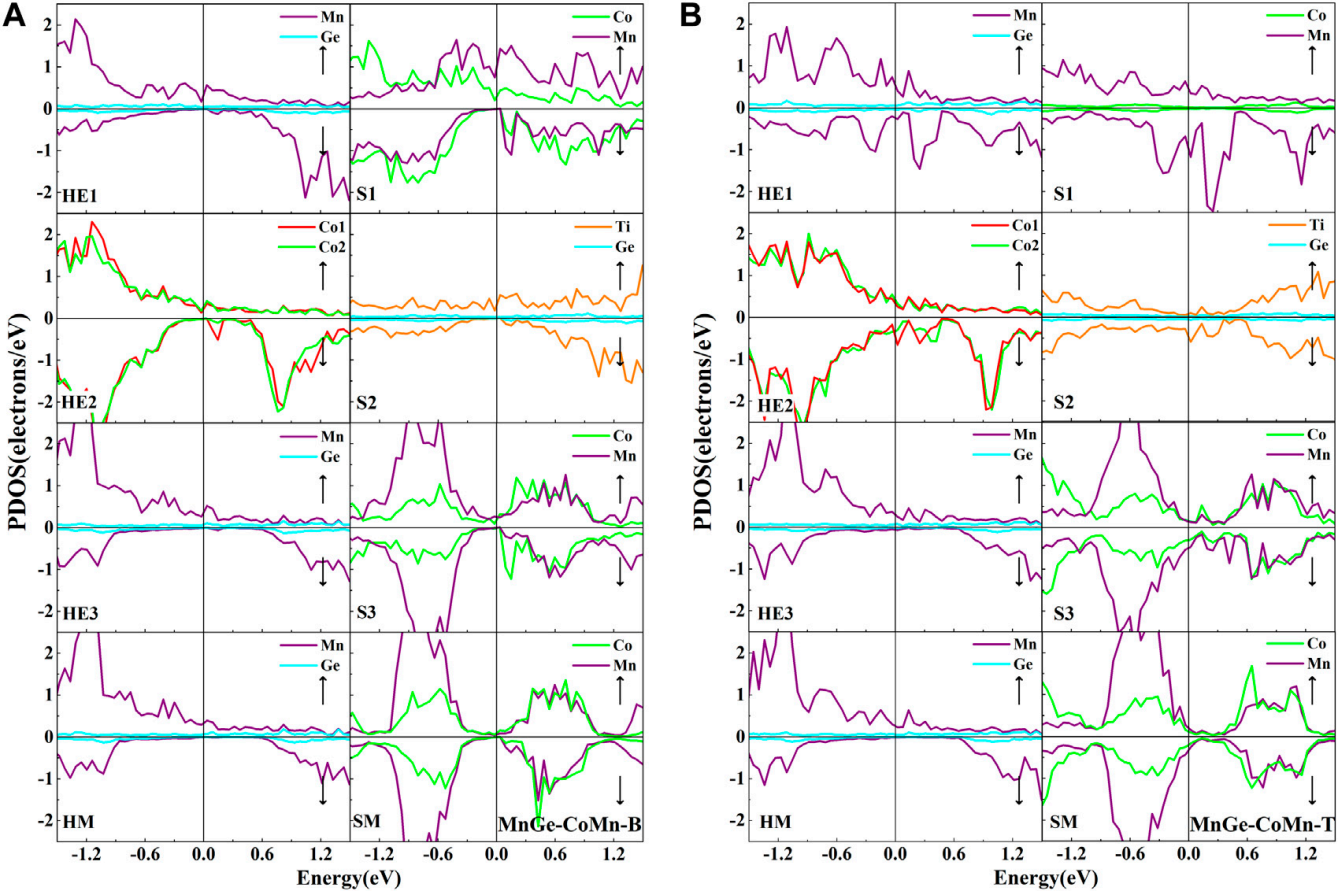
Atomic PDOS of the interface (HE1), the subinterface layer (HE2), the next subinterface layer (HE3), and the middle layer (HM) in the Co_2_MnGe slab and the interface layer (S1), the subinterface layer (S2), the next interface layer (S3), and the middle layer (SM) in the CoTiMnGe slab of **(A)** MnGe-CoMn-B and **(B)** MnGe-CoMn-T heterojunctions.

For the MnGe-CoMn-B heterojunction, as shown in Figure 8A, several interface peaks appear near the Fermi level from the three outermost layer atoms. Although the results show up to 97.75% spin-polarization value in the II-type structure, all half-metallic or semiconductor gaps were severely shortened in both the Co_2_MnGe and CoTiMnGe slabs. Some environmental factors, such as thermal excitation, illumination, and defects, may cause large depolarization of the system. For the MnGe-CoMn-T heterojunction, as shown in Figure 8B, because of the destruction of the interface states from the Co atoms at the HE2 layer, Mn atoms at the S1 layer, and Ti atoms at the S2 layer, the spin-polarization value of the II-type structure did not exceed 1%.

**FIGURE 9:**
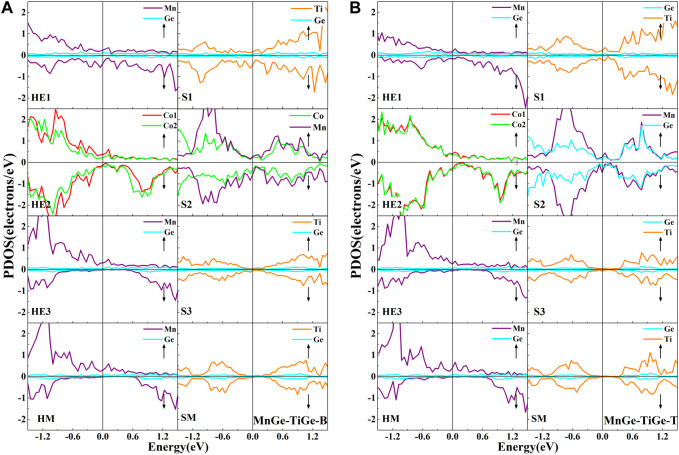
Atomic PDOS of the interface (HE1), the subinterface layer (HE2), the next subinterface layer (HE3), and the middle layer (HM) in the Co_2_MnGe slab and the interface layer (S1), the subinterface layer (S2), the next interface layer (S3), and the middle layer (SM) in the CoTiMnGe slab of **(A)** MnGe-TiGe-B and **(B)** MnGe-TiGe-T heterojunctions.

In the end, for the MnGe-TiGe-B and MnGe-TiGe-T heterojunctions, as shown in Figure 9 and Table 5, a significant depolarization caused by Mn atoms at the HE1 layer and Co atom at the HE2 layer resulted in a spin-polarization value of less than 14% in both heterojunctions.

## 4 Conclusion

Based on first-principles calculations in DFT methods, we used the GGA + PBE scheme to comprehensively investigate the interface structural effect, atomic displacement effect, atomic magnetism, and electronic properties of the eight possible interface structures in the all-Heusler alloy Co_2_MnGe/CoTiMnGe(100) heterojunction. The calculated band structures revealed that Co_2_MnGe is a typical half-metal material with a half-metallic gap of approximately 0.435 eV. CoTiMnGe is a narrow-band-gap semiconductor with a band gap width of 0.2 eV. It utilizes a wide range of solar light and may act as an ultra-sensitive photocatalyst to decompose harmful substances. Their equilibrium lattices are 5.742 Å and 5.817 Å, respectively. Hence, the lattice mismatch ratio of the Co_2_MnGe/CoTiMnGe heterojunction is less than 0.65%. Direct magnetic hybridization and indirect RKKY exchange, influenced by the surrounding atomic environment, are crucial for the magnetic properties of atoms in both the Co_2_MnGe(100) surface and the Co_2_MnGe/CoTiMnGe(100) heterojunction. For eight possible Co_2_MnGe/CoTiMnGe(100) heterojunctions, the half-metallic gaps in the Co_2_MnGe bulk have been destroyed by the inevitable interface states. For the spin-polarization ratio, although the MnGe-CoMn-B heterojunction shows up to 97.75% spin-polarization value in the II-type structure, all half-metallic or semiconductor gaps were severely shortened in both the Co_2_MnGe and CoTiMnGe slabs. In the CoCo-TiGe-B heterojunction, we observe that the half-metallic properties in the bulk are preserved for the Co atom at the HE1 and the Mn atom at the HE2 layer. The semiconductor gap in the CoTiMnGe bulk is also retained. It is feasible to search for high-performance MTJs by artificially constructing suitable all-Heusler alloy heterojunctions.

## Data Availability

The original contributions presented in the study are included in the article/Supplementary Material; further inquiries can be directed to the corresponding authors.
